# Analysis of GSDMD-N abnormality promoting neutrophil NETs mediated RA disease through NLRP3-dependent pathway

**DOI:** 10.3389/fimmu.2025.1652608

**Published:** 2025-09-29

**Authors:** Yali Sang, Huiyang Liu, Bingle Li, Lingyan Zhu, Yongfu Wang, Li Bai

**Affiliations:** ^1^ The First Affiliated Hospital of Baotou Medical College, Inner Mongolia University of Science & Technology, Baotou, China; ^2^ Department of Rheumatology and Immunology, the First Affiliated Hospital of Baotou Medical College, Baotou, China; ^3^ The Central Lab, the First Affiliated Hospital of Baotou Medical College (Inner Mongolia Autoimmune Key Laboratory), Baotou, China

**Keywords:** rheumatoid arthritis, NLRP3 inflammasome, neutrophils, NEtosis, GSDMD

## Abstract

**Introduction:**

Rheumatoid arthritis (RA) is characterized by persistent synovitis and progressive joint damage. Mounting evidence implicates neutrophil extracellular traps (NETs) formation (NETosis) in RA pathogenesis, yet the upstream regulatory nodes remain incompletely defined. We aimed to elucidate the role of the NLRP3 inflammasome in regulating GSDMD-dependent NETosis and to evaluate whether inhibiting NLRP3 or Gasdermin D (GSDMD) alleviates RA pathology.

**Methods:**

Neutrophils and synovial tissues from patients with RA and osteoarthritis (OA) were analyzed for NLRP3, GSDMD, and NET-related markers by immunofluorescence, Western blot, and qPCR. A collagen-induced arthritis (CIA) mouse model was used to test the effects of pharmacological inhibition of NLRP3 or blockade of GSDMD pore formation on joint swelling, bone destruction, and synovial inflammation. Transcriptomic sequencing was performed to identify differentially expressed genes following inhibition of GSDMD pore formation. Ionomycin was used to induce ROS-independent NETosis in vitro.

**Results:**

The RA group exhibited elevated NLRP3/GSDMD expression and increased NET markers relative to OA controls. In CIA mice, inhibition of NLRP3 or blockade of GSDMD pore formation mitigated joint swelling, reduced bone erosion, and attenuated synovial inflammation. Transcriptomic profiling identified 12 core genes—enriched for heat-shock proteins and histone variants—with significant differential expression after GSDMD pore inhibition. Ionomycin stimulation enhanced ROS-independent NET formation and was accompanied by increased NLRP3 expression and promoted cleavage of GSDMD into its active N-terminal fragment (GSDMD-N). Conversely, suppressing NLRP3 activation or preventing GSDMD pore formation effectively inhibited this process.

**Discussion:**

These data position the NLRP3 inflammasome as a pivotal upstream regulator of GSDMD-dependent NETosis in RA. Targeting this axis—via inhibition of NLRP3 or blockade of GSDMD pore formation—ameliorates inflammatory joint pathology in vivo and constrains NETosis in vitro. Collectively, our findings support the NLRP3–GSDMD pathway as a promising therapeutic avenue for RA.

## Graphical Abstract

Pathways of NLRP3 inflammasome activation and ROS-independent NETosis. Left panel: A two-signal model primes (NF-κB–dependent upregulation of NLRP3 and pro-cytokines) and activates NLRP3, which recruits ASC to activate caspase-1; caspase-1 cleaves GSDMD, whose N-terminal pores enable IL-1β/IL-18 maturation and release, initiating pyroptosis-associated inflammation. Right panel: Under ROS-independent conditions, ionomycin-induced Ca^2+^ influx activates PAD4, driving histone citrullination and chromatin decondensation, which synergize to cause nuclear envelope breakdown and the extrusion of DNA web-like structures (NETs). Created in BioRender. Liu, R. (2025) https://BioRender.com/hz7utj2, with permission from Liu, R.

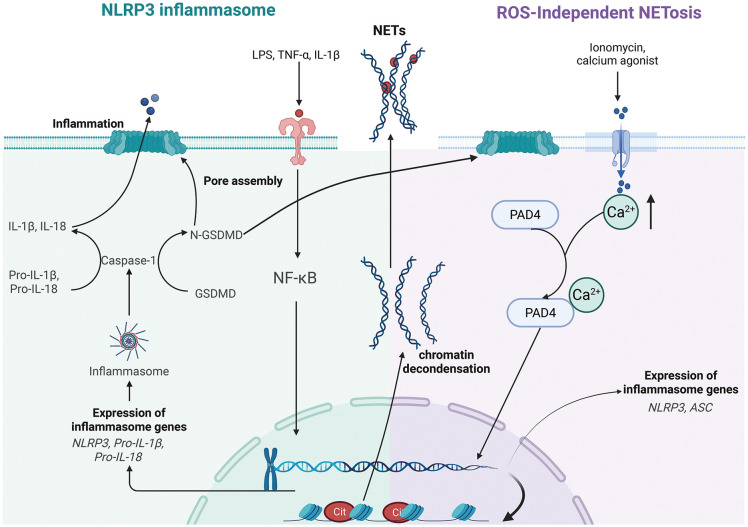

## 1 Introduction

Rheumatoid arthritis (RA) is a chronic inflammatory disorder characterized by inflammation of the synovial membranes and progressive joint destruction. Recent research has highlighted that the formation of neutrophil extracellular traps (NETs)—a process known as NETosis—plays a pivotal role in RA pathogenesis. NETosis is a specialized form of programmed cell death in which neutrophils release a mesh-like structure composed of DNA and antimicrobial proteins to trap and kill pathogens. However, when NETosis is excessively activated and large amounts of NETs are released, it can lead to sustained inflammation and has been implicated in various inflammatory conditions, autoimmune disorders, cardiovascular diseases, and tumor metastasis (1–4). Furthermore, studies indicate that neutrophils in both the peripheral blood and synovial fluid of RA patients are more prone to undergo NETosis, and the levels of NETs correlate closely with disease activity (1). The components of NETs, including histones, DNA, and other proteins, contribute to the generation of autoantigens that drive autoimmune responses, such as anti-citrullinated protein antibodies (5). Moreover, the abnormal increase in neutrophil NETs in RA patients is also considered to be related to delayed neutrophil apoptosis and dysfunction in their migration (6). Taken together, these findings suggest that excessive NET release, through its role in perpetuating autoimmunity and maintaining an inflammatory microenvironment, is central to the pathological progression of RA.

In recent years, members of the Gasdermin (GSDM) family, particularly Gasdermin D (GSDMD), have been identified as key regulatory proteins in NETosis. GSDMD is a critical executioner protein in pyroptosis. Once activated, GSDMD is cleaved into its active N-terminal fragment (GSDMD-N) and inactive C-terminal fragment (GSDMD-C). The active GSDMD-N can form pores approximately 20 nm in diameter in the plasma membrane, increasing the membrane’s permeability, leading to the leakage of cellular contents, and triggering pyroptosis (7, 8). Research by Karmakar et al. suggests that, unlike pore formation in macrophage membranes, GSDMD-N can form pores in the membranes of neutrophil azurophilic granules, promoting the release of myeloperoxidase (MPO), neutrophil elastase (NE) (9), and DNA (10). As membrane pores form and cell membrane integrity is disrupted, the chromatin and antimicrobial proteins released by neutrophils further aggregate to form NETs. Research by Sollberger et al. has shown that membrane pore formation mediated by GSDMD-N induces the release of neutrophil granules and chromatin decondensation, which are necessary steps for NET formation (10).

As a key executioner protein in pyroptosis, the activation of GSDMD is closely associated with inflammasomes. The most widely studied inflammasome in RA is the NLRP3 inflammasome. The NLRP3 inflammasome consists of three main components: the sensor protein NLRP3, the adaptor protein ASC (Apoptosis-associated speck-like protein containing a CARD), and the effector protein caspase-1 (11). Activation of NLRP3 recruits ASC, which in turn recruits and activates caspase-1. Activated caspase-1 cleaves the precursor forms of pro-inflammatory cytokines such as Pro-IL-1β and Pro-IL-18, releasing them extracellularly and triggering strong inflammatory responses (12). Activation of the NLRP3 inflammasome requires two signals: the first signal (priming signal) upregulates NLRP3 and pro-inflammatory cytokine expression through the NF-κB pathway, while the second signal (activating signal) directly triggers NLRP3 inflammasome assembly and activation (13).

Several studies have pointed to the important role of the NLRP3 inflammasome in RA (14) (15). NLRP3 can sense danger signals both inside and outside the cell, such as urate crystals, mitochondrial ROS, extracellular ATP, and cartilage fragments. This leads to the activation of various cell types, including synovial fibroblasts (FLS), macrophages, T cells, and monocytes, which undergo pyroptosis (16), ultimately contributing to synovitis and joint damage. However, the function of the NLRP3 inflammasome in neutrophils remains unclear. Research by Dhruv Chauhan et al. suggests that activation of the classical NLRP3 inflammasome causes pyroptosis but does not induce NETosis (17). Studies by Kaiwen W. Chen et al. propose that compared to macrophages, neutrophils express low levels of caspase-1 and ASC, and activation of the classical NLRP3 inflammasome does not induce neutrophil pyroptosis in order to preserve host defense functions (18). A study by Patrick Münzer et al. suggests that inflammasome-dependent signaling in inflammatory diseases is part of the process that drives NETosis (19).

RA is an autoimmune disease characterized by chronic synovitis and joint destruction, and its progression is closely related to NETosis. Excessive activation of NETosis leads to the accumulation of NETs, the release of histones, DNA, and other components, inducing autoimmune responses and exacerbating inflammation. GSDMD can be cleaved into the active form, GSDMD-N, which forms membrane pores in neutrophils, promoting the release of MPO, NE, and chromatin, driving the formation of NETs. The NLRP3 inflammasome is widely activated in RA, responding to danger signals through pathways such as NF-κB, promoting the release of IL-1β/IL-18 and triggering pyroptosis, which exacerbates synovitis. However, the relationship between the NLRP3 inflammasome and NETosis in neutrophils remains controversial. Some studies suggest that NLRP3 activation primarily induces pyroptosis, while neutrophils may participate in NET formation through a non-canonical pathway mediated by GSDMD. The synergistic or independent regulatory mechanisms between GSDMD-mediated NETosis and the NLRP3 inflammasome in RA need further exploration to clarify their dynamic roles in disease progression.

## 2 Materials and methods

### 2.1 Animals and reagents

Mice (DBA1, 6 weeks old, 20–25 g) were purchased from the Nanjing Model Animal Center. All experimental procedures were reviewed and approved by the Ethics Committee of the First Affiliated Hospital of Baotou Medical College and conducted in accordance with the Guide for the Care and Use of Laboratory Animals (approval number D044). Disulfiram (Lemaitan Pharmaceuticals) was used as a GSDMD inhibitor, and MCC950 (MCE) was used as an NLRP3 inhibitor; the BCA protein assay kit was acquired from Beyotime Biotechnology; anti-GSDMD and anti-GSDMD-N antibodies were purchased from Proteintech; and the HRP-conjugated anti-rabbit secondary antibody was obtained from Abcam.

### 2.2 Construction of CIA mice and joint swelling score

To establish the CIA (collagen-induced arthritis) mouse model, 100 μL of Mycobacterium tuberculosis solution (4 mg/mL) emulsified with 100 μL of type II collagen in Freund’s adjuvant was intravenously injected into the tail base (2 cm from the root) of DBA/1 mice. On day 21, a booster immunization was administered using the same procedure. Mice were randomly assigned into experimental groups as follows. For the first set of experiments, animals were randomly divided into three groups (n = 6 per group): Control, CIA, and CIA treated with Disulfiram (50 mg/kg, intraperitoneally, once daily). For the second set of experiments, animals were randomly divided into four groups (n = 6 per group): Control, CIA, and CIA treated with MCC950 (50 mg/kg, intraperitoneally, every other day). The severity of joint swelling was then scored, followed by sacrificing the mice and isolating the hind limbs for radiological evaluation. Micro-computed tomography (micro-CT) images of the hind limbs were acquired using a high-resolution *in vivo* X-ray cone beam scanner. Histological assessments were performed using hematoxylin-eosin (HE) staining and toluidine blue (TB) staining.

Joint pathology in CIA mice was assessed using HE and TB staining. For HE staining, inflammation, synovial hyperplasia, cartilage destruction, and bone erosion were each graded on a 0–3 scale, with higher scores indicating greater severity (0 = normal, 3 = severe damage with extensive infiltration or tissue loss). For TB staining, proteoglycan content and cartilage structure were evaluated: proteoglycan loss was scored from 0 (uniform deep blue, no loss) to 4 (complete loss of staining), and structural damage was scored from 0 (intact surface) to 4 (deep fissures reaching subchondral bone). All sections were scored independently by two blinded observers, and mean values were used for analysis.

### 2.3 Isolation of neutrophils

Patients with OA and RA who visited the First Affiliated Hospital of Baotou Medical College between September 1, 2023, and April 1, 2025, were enrolled in this cohort study. Baseline information was obtained from electronic medical records (**Supplementary Table 1**), and the inclusion and exclusion criteria are detailed in **Supplementary Table 2**. This study was approved by the Ethics Committee of the First Affiliated Hospital of Baotou Medical College (approval number K126-01) and was conducted in accordance with the principles of the Declaration of Helsinki.

Peripheral blood (8 mL) from OA or RA patients was collected in sodium citrate or EDTA anticoagulant tubes and mixed well. Neutrophils were isolated using the EasySep™ Neutrophil Isolation Kit. The EasySep reagent was added to the sample, mixed, and incubated with magnetic beads to bind the target cells. The sample tube was placed in a magnetic separator, where the magnetic beads (non-target cells) were adsorbed to the tube wall, and the target cells remained in the solution. The solution containing the target cells was transferred to a new tube to obtain highly purified neutrophils.

### 2.4 Immunofluorescence staining for NET formation

Peripheral blood neutrophils were seeded onto 24-well plates. In the experimental groups, neutrophils were treated with Disulfiram (50 μmol/L) or MCC 950 (10 μmol/L) along with ionomycin (5 μmol/L) for 2 hours. The cells were fixed with 4% paraformaldehyde for 30 minutes, followed by blocking with 5% bovine serum albumin (BSA) at room temperature for 30 minutes. The primary antibodies were then added, and the cells were incubated overnight at 4°C. Afterward, the corresponding secondary antibodies were added, and cells were incubated for 1 hour in the dark. The cells were then washed three times with PBST and stained with DAPI for 10 minutes to visualize the nuclei. NETs and NLRP3/GSDMD protein expression were observed under a laser confocal microscope, and analysis was performed using ImageJ.

### 2.5 Western blot analysis

Neutrophils were collected and lysed with RIPA buffer containing PMSF at a 100:1 ratio to extract proteins. The extracted proteins were loaded into 10% SDS-PAGE gels and electrophoresed. After electrophoresis, proteins were transferred to a PVDF membrane, which was blocked with BSA at room temperature for 1 hour. The membrane was incubated with specific primary antibodies overnight at 4°C. The next day, corresponding secondary antibodies were added, and the membrane was incubated for 1 hour at room temperature. Protein bands were visualized using Supersignal™ West Femto Ultrasensitive Substrate and analysis was performed using ImageJ.

### 2.6 RNA extraction from neutrophils

RNA was extracted from neutrophils using the TRIzol method. First, TRIzol reagent was added to lyse the cells. After adding chloroform and vigorous shaking for 15 seconds, the mixture was left standing for 5 minutes and then centrifuged at 12,000 g for 15 minutes. The supernatant was transferred to a new tube, and an equal volume of isopropanol was added. The mixture was incubated at -20°C for 10 minutes and then centrifuged at 12,000 g for 10 minutes to precipitate RNA. The RNA pellet was washed twice with 75% ethanol, dissolved in RNase-free water, and stored at -80°C for future use. RNA purity was assessed using a NanoDrop 2000 spectrophotometer, and samples with an A260/A280 ratio between 1.8 and 2.0 were selected for further experiments.

### 2.7 Transcriptomic sequencing and protein-protein interaction network analysis

Peripheral blood neutrophils were isolated from RA patients and treated with ionomycin or disulfiram(n=4 per group), followed by transcriptomic sequencing. Raw sequencing data were subjected to quality control, during which low-quality reads, adaptor sequences, and reads containing excessive unknown bases (N) were removed using FastQC and Trimmomatic. Clean reads were then assessed for quality metrics, including base quality score (Q20/Q30), GC content, and sequence duplication levels, to ensure data reliability. After quality control, differential expression analysis was performed to identify differentially expressed genes (DEGs).

Significantly differentially expressed protein-coding genes (|log_2_FoldChange| ≥ 2, p < 0.05) were extracted from the RNA-Seq data, and a protein–protein interaction (PPI) network was constructed using the STRING v11.5 database. Hub genes were further identified using Cytoscape software, applying three topological algorithms (MCC, DMNC, and MNC). The intersection of the top 20 genes obtained from each method was defined as the hub gene set. Finally, Gene Ontology (GO) enrichment and Kyoto Encyclopedia of Genes and Genomes (KEGG) pathway enrichment analyses were conducted to investigate the biological processes and signaling pathways associated with these hub genes.

### 2.8 Statistical analysis

Statistical analysis was performed using GraphPad Prism 9.0. All experimental data were expressed as mean ± standard deviation (Mean ± SD). Data with normal distribution were analyzed using parametric tests, and non-parametric tests were used when data did not meet the normality assumption. For comparisons between two groups, t-tests were used. For comparisons among multiple groups, one-way ANOVA was performed, followed by Tukey’s multiple comparison test for pairwise significance analysis. For non-normally distributed data, the Mann-Whitney U test (for two groups) or Kruskal-Wallis H test (for multiple groups) was used. A p-value of < 0.05 was considered statistically significant, with significance levels marked as follows: *p < 0.05, **p < 0.01, ***p < 0.001. Each experiment was independently repeated at least three times.

## 3 Results

### 3.1 Increased neutrophil infiltration in synovium and enhanced neutrophil extracellular traps formation in peripheral blood of RA patients

**Figure 1:**
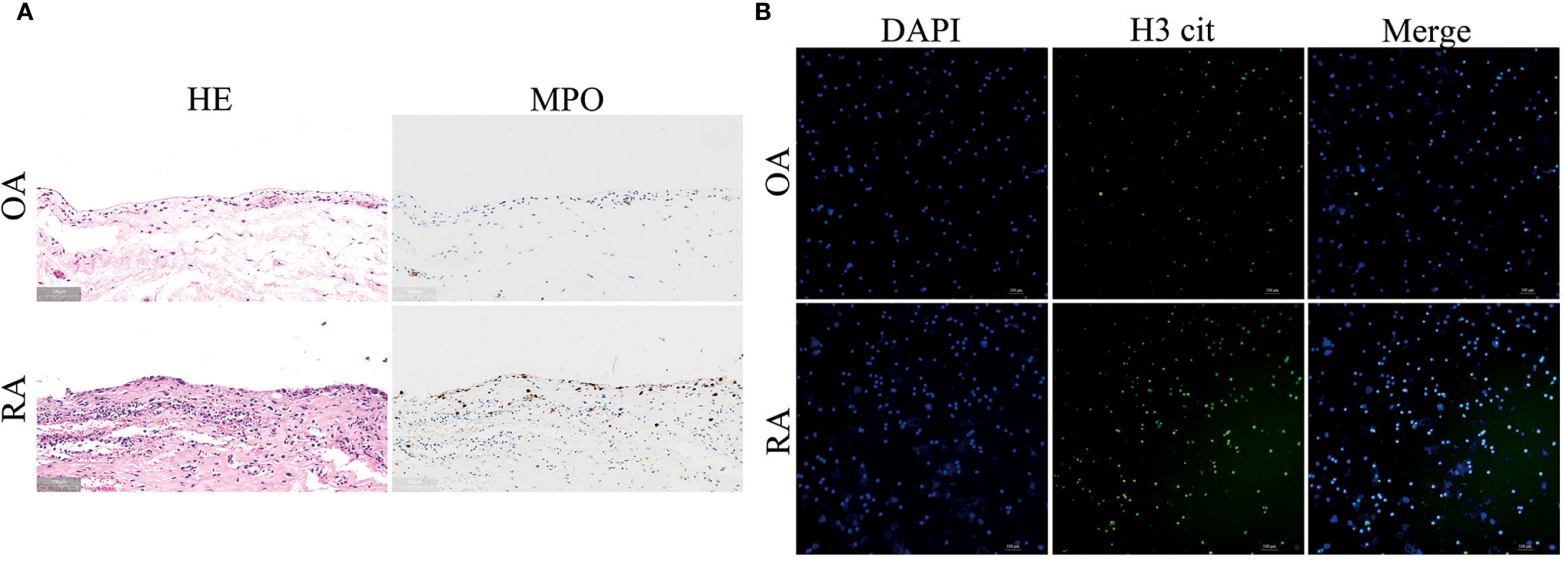
Increased neutrophil infiltration in synovium and enhanced neutrophil extracellular traps (NETs) formation in peripheral blood of RA patients. **(A)** HE staining (left panel) and MPO immunohistochemical staining (right panel) of synovial tissues from RA and OA patients. **(B)** Immunofluorescence staining of NETs formation in peripheral blood neutrophils from RA and OA patients. DAPI (blue) labels nuclei, and H3 cit (green) marks citrullinated histone H3.

HE staining images showed that, compared to the OA group, the synovial cells in the RA group were shed, synovial fibroblasts were proliferating, and inflammatory cell infiltration was increased. MPO staining images showed that neutrophil infiltration in the synovium of RA patients was higher (**Figure 1A**). To verify the abnormal function of neutrophils, we performed immunofluorescence staining on neutrophils from the peripheral blood of both OA and RA patients. It was observed that, compared to the OA group, neutrophils in the RA group exhibited significant NET release, with an increase in citrullinated histone H3 (H3cit) content (**Figure 1B**). These results suggest that there is increased neutrophil infiltration in the synovium of RA patients, and abnormal neutrophil function is evident in the peripheral blood.

### 3.2 GSDMD promotes neutrophil NET release, exacerbating rheumatoid arthritis progression; inhibition of GSDMD alleviates arthritis inflammation and damage

**Figure 2:**
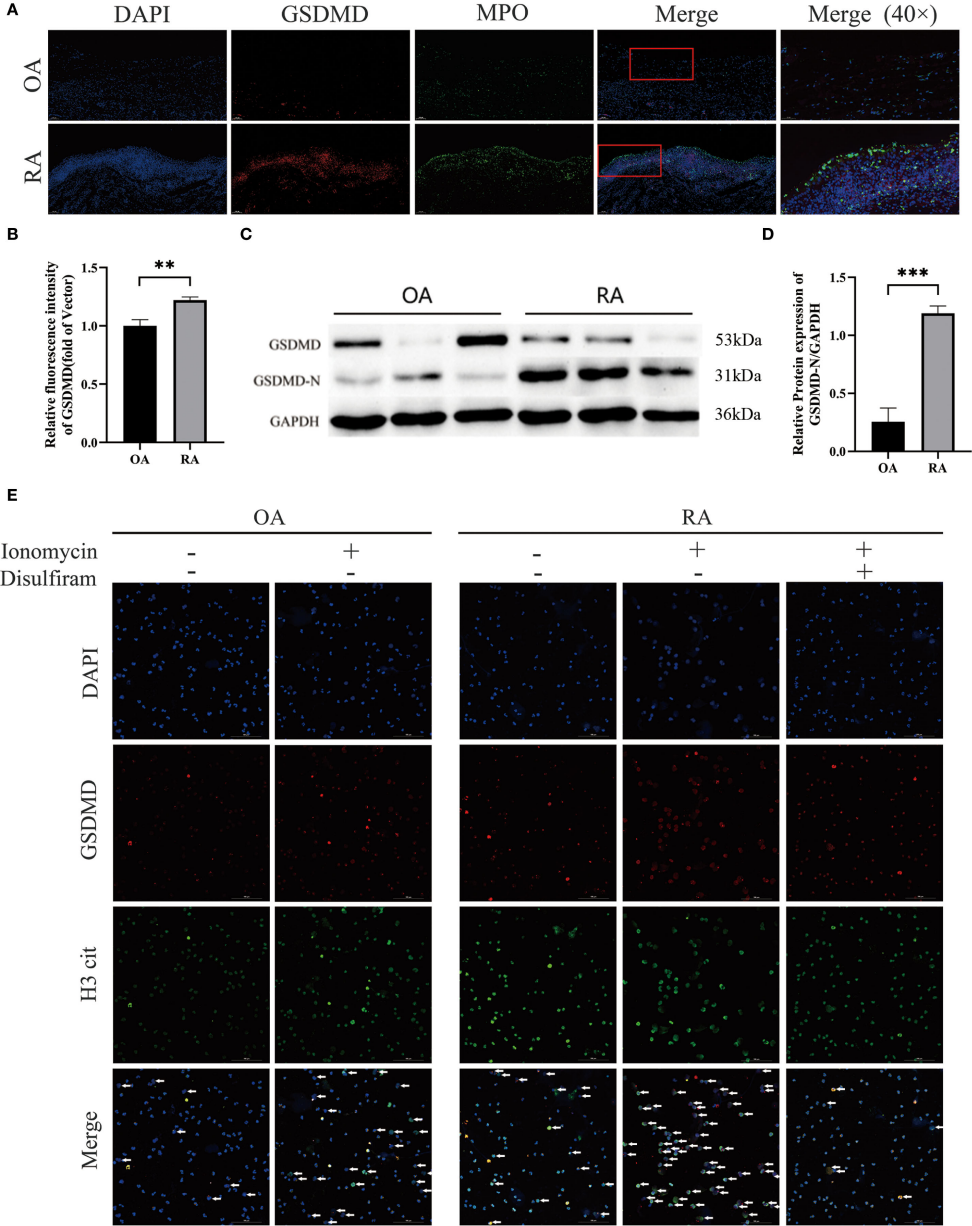
Elevated GSDMD expression in synovial and peripheral-blood neutrophils from patients with rheumatoid arthritis (RA). **(A)** GSDMD and its co-localization with MPO in synovial tissues from OA and RA patients. **(B)** Quantitative immunofluorescence analysis of GSDMD expression in the synovial tissues of both groups of patients. **(C, D)** Western blot analysis of GSDMD-N protein expression in peripheral blood neutrophils from OA and RA patients. **(E)** Confocal microscopy was used to examine NETs formation and GSDMD expression in peripheral blood neutrophils from OA and RA patients following treatment with Ionomycin (5 μmol/L) and Disulfiram (50 μmol/L). Arrows indicate cells undergoing NETs formation. ** p < 0.01, *** p < 0.001.

Immunofluorescence imaging showed that the expression of GSDMD in the synovial tissue of RA patients was elevated compared to that in the OA group (**Figure 2A**) and co-localized with MPO (**Figure 2B**). In addition, Western blot analysis (**Figures 2C, D**) revealed that GSDMD activation in neutrophils from RA patients was higher than in those from OA patients. To investigate the effect of GSDMD on NETs formation in neutrophils, we treated peripheral blood neutrophils from both OA and RA groups with ionomycin and performed immunofluorescence staining to assess NETs release. A significant increase in NETs release was observed in both groups after ionomycin stimulation, while treatment with disulfiram, a GSDMD inhibitor, significantly reduced NETs formation (**Figure 2E**). These results indicate that inhibition of the pore-forming activity of GSDMD reduces NETs secretion in neutrophils from RA patients.

**Figure 3:**
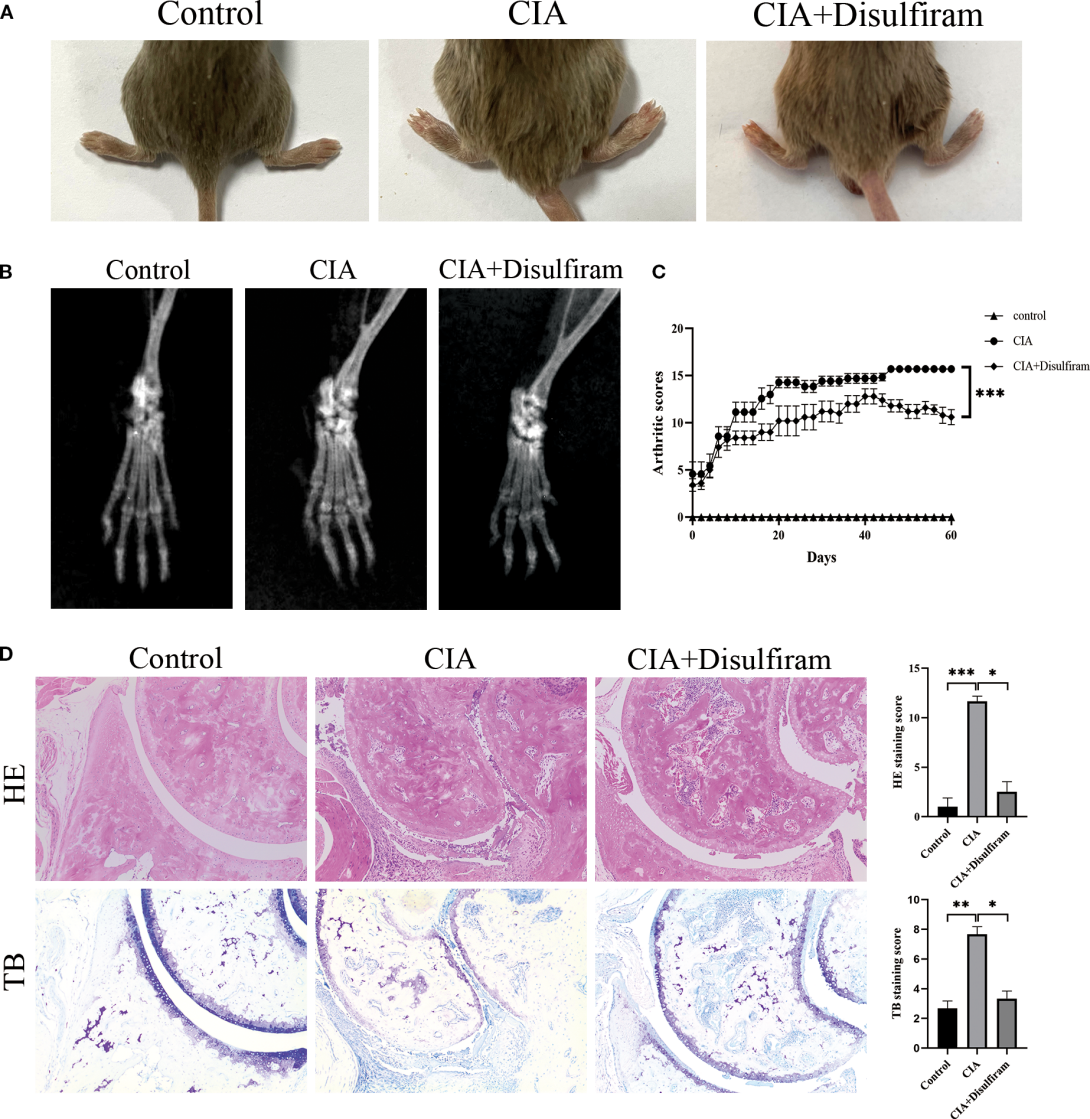
Disulfiram-mediated GSDMD inhibition alleviates collagen-induced arthritis (CIA) in mice. Mice received intraperitoneal injections of disulfiram (50 mg /kg/day per animal). **(A)** Representative photographs of hind paws from each group on day 60 of treatment. Arthritis severity was graded on a 0–4 scale. Data for the control, CIA, and disulfiram-treated groups are presented as mean ± SD (n = 6). **(B)** Micro-computed tomography (micro-CT) cross-sections of hind-ankle joints after 60 days of treatment, used to assess bone destruction. **(C)** Arthritis scores of the mice. **(D)** HE and TB staining, along with histological scoring, were performed on ankle-joint sections to evaluate synovial pathology and bone erosion/destruction. * p < 0.05, ** p < 0.01, *** p < 0.001.

To verify the role of GSDMD in RA progression, we performed *in vivo* experiments using a collagen-induced arthritis (CIA) mouse model. After intervention with Disulfiram (GSDMD inhibitor) in CIA mice, we found that joint swelling and arthritis scores were significantly reduced in the Disulfiram-treated group (**Figures 3A, B**). Radiographic evaluation of the mouse ankle joints showed that CIA mice exhibited significant joint space narrowing and reduced bone density, while Disulfiram treatment improved these radiographic features (**Figure 3C**). HE and TB staining results also showed that in CIA mice, there was increased infiltration of inflammatory cells in the synovium, along with connective tissue proliferation covering the cartilage surface, and evidence of cartilage and subchondral bone erosion. After Disulfiram treatment, the inflammatory cell infiltration decreased, and cartilage and subchondral bone erosion was alleviated (**Figure 3D**). These results collectively suggest that inhibiting GSDMD can alleviate arthritis symptoms and joint damage in CIA mice.

### 3.3 GSDMD regulates neutrophil NET release, and inhibition of GSDMD-N pore formation is associated with heat shock proteins and histone-related hub genes

**Figure 4:**
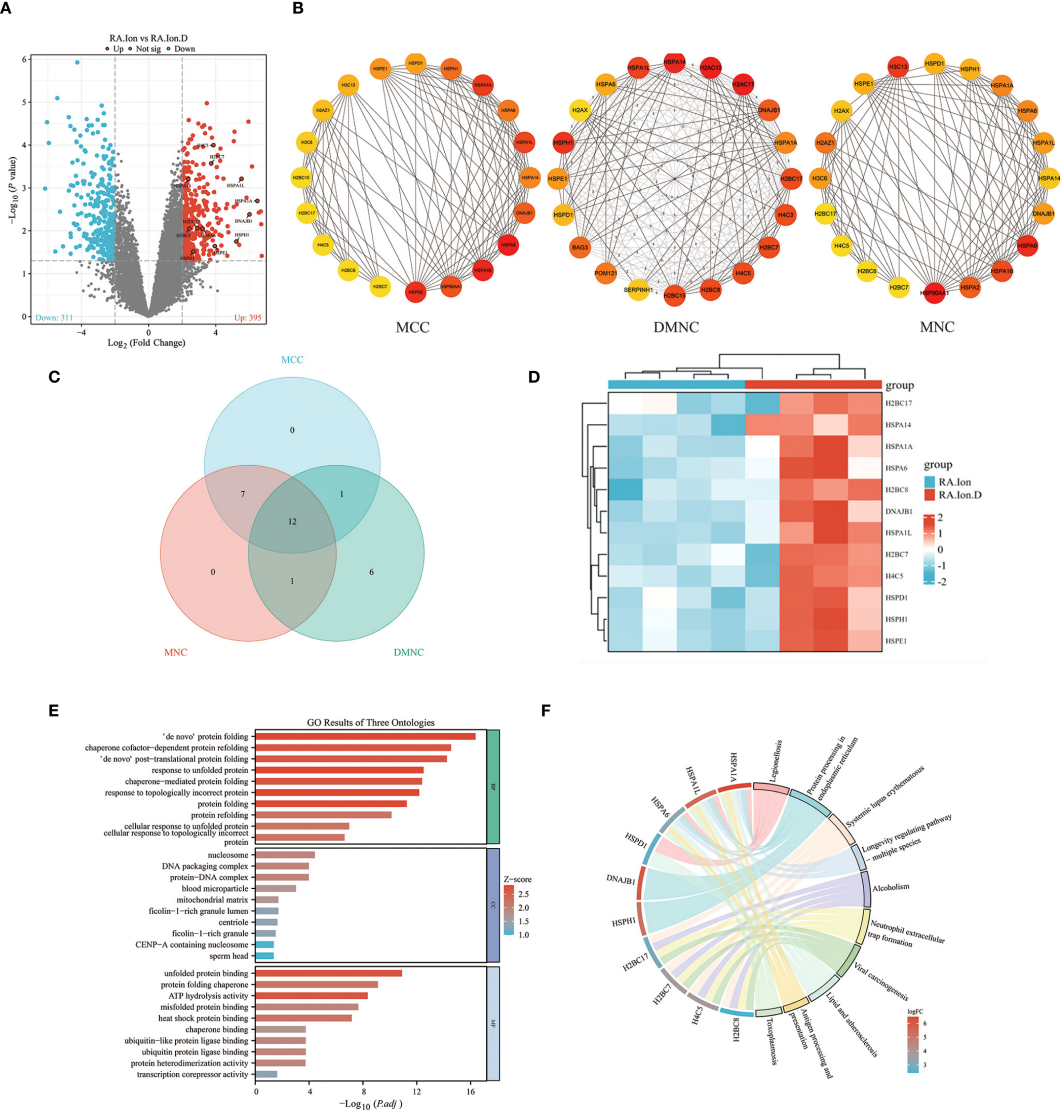
Transcriptomic analysis. **(A)** Volcano plot displaying the distribution of differentially expressed genes (DEGs) between the two groups. **(B, C)** Hub-gene screening and Venn diagram: key genes and their interactions were identified with three independent algorithms, yielding 12 overlapping hub genes. **(D)** Heat-map showing the expression levels of these 12 hub genes in the two groups. **(E)** Gene Ontology (GO) enrichment analysis indicates that the 12 hub genes are concentrated in biological processes such as neutrophil activation, immune response, and degranulation. **(F)** KEGG enrichment analysis reveals that the same gene set is over-represented in immunity- and inflammation-related pathways, including cytokine–cytokine receptor interaction, chemokine signalling, TNF signalling, and the NETosis pathway.

To further explore the role of GSDMD in RA pathogenesis, we performed transcriptomic analysis on peripheral blood neutrophils from RA patients and treated with ionomycin or disulfiram, and divided into the RA.Ion group and the RA.Ion.D group group. The volcano plot displayed 706 differentially expressed genes (DEGs), with 395 upregulated genes and 311 downregulated genes (**Figure 4A**). We identified key genes through MCC, DMNC, and MNC methods, and conducted co-expression network analysis, resulting in the identification of 12 core genes (**Figures 4B, C**). A heatmap of differential gene expression further revealed the gene expression patterns between the RA.Ion group and the RA.Ion.D group. Hub genes were screened from the transcriptomic data, and enrichment analysis showed that Disulfiram intervention affected 12 key genes involved in neutrophil activation, immune response, and degranulation (**Figure 4D**).

**Figure 5:**
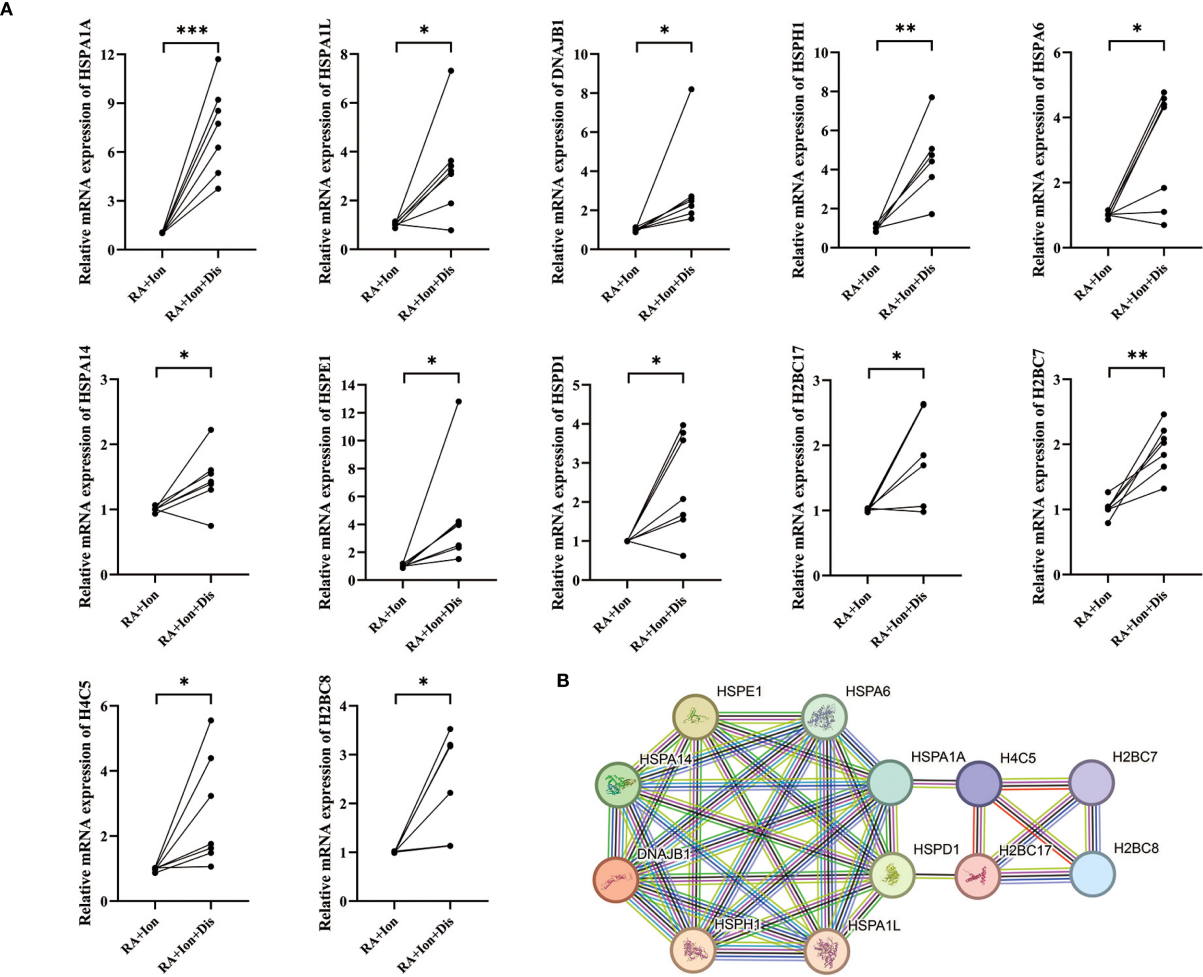
Validation of differentially expressed genes (DEGs) by qPCR and protein-interaction network analysis. **(A)** RT-qPCR validation of DEGs. Relative mRNA levels of 12 DEGs—HSPA1A, HSPA1L, DNAJB1, HSPH1, HSPA6, HSPA14, HSPE1, HSPD1, H2BC17, H2BC7, H4C5, and H2BC8—were compared between the two experimental groups. **(B)** Protein–protein interaction (PPI) network. Each node represents a protein encoded by the 12 validated DEGs, and edges denote predicted functional or physical interactions between these proteins. * p < 0.05, ** p < 0.01, *** p < 0.001.

GO enrichment analysis revealed that these genes were primarily enriched in biological processes related to neutrophil activation, immune responses, and degranulation (**Figure 4E**). KEGG pathway enrichment analysis showed that these genes were significantly enriched in immune or inflammatory-related pathways, including cytokine-cytokine receptor interaction, chemokine signaling pathways, and TNF signaling pathways. Additionally, Disulfiram intervention was found to influence NET release (**Figure 4F**). Based on these results, we speculated that the inhibition of GSDMD pore formation could affect NET release and that Disulfiram-mediated GSDMD inhibition might involve these 12 genes. qPCR validation of these 12 genes showed consistent results with the sequencing data (**Figure 5A**). To explore the relationships between these genes, we conducted protein-protein interaction (PPI) network analysis, which indicated potential interactions between HSPA1A and H4C5, as well as HSPD1 and H2BC17 (**Figure 5B**).

### 3.4 Aberrant activation of NLRP3 inflammasome in RA neutrophils and synovium and co-localization with neutrophil infiltration in synovium

**Figure 6:**
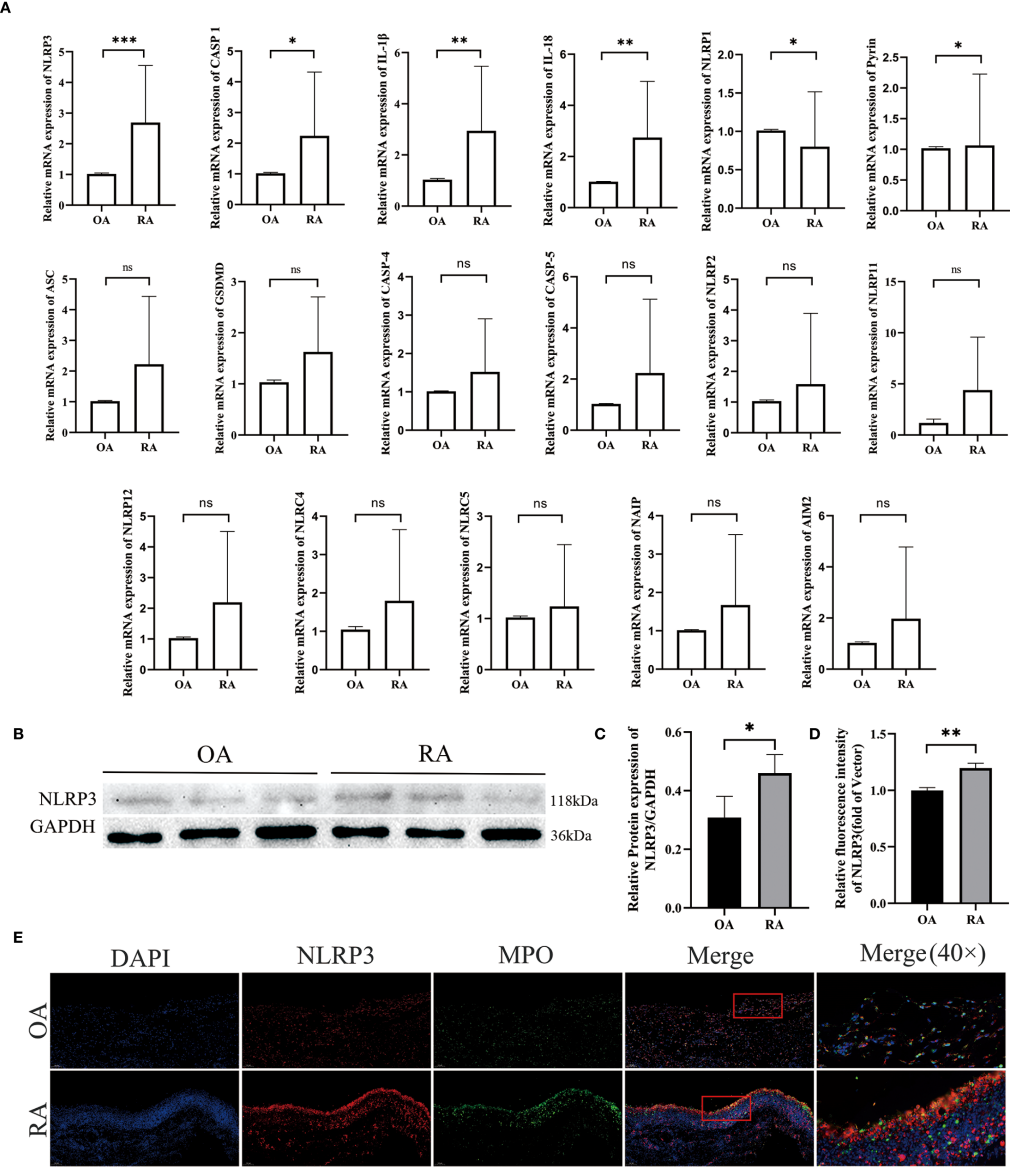
Co-localization of aberrantly activated inflammasome gene NLRP3 in RA neutrophils with synovial inflammatory infiltration. **(A–C)** Differences in inflammasome activity between peripheral-blood neutrophils from rheumatoid-arthritis (RA) patients and osteoarthritis (OA) controls were assessed. qPCR: mRNA levels of inflammasome-related genes were measured, with GAPDH as the internal reference. Western blot: NLRP3 protein expression was detected and quantified. **(D)** Quantitative immunofluorescence analysis of NLRP3 expression in synovial tissues from OA and RA patients. **(E)** Immunofluorescence double-staining of synovial tissues from RA and OA patients revealed co-localization of NLRP3 and MPO. * p < 0.05, ** p < 0.01, *** p < 0.001, ns, not significant.

To investigate the expression of inflammasomes in neutrophils from RA patients, we collected peripheral blood neutrophils from both OA and RA patients and analyzed the expression of inflammasome-related genes using qPCR. The results showed that, compared to the OA group, the gene expression levels of NLRP3, CASP1, IL-1β, IL-18, and Pyrin were upregulated, while NLRP1 was downregulated. No significant differences were observed between the two groups in the expression of ASC, GSDMD, CASP4, and CASP5 (**Figure 6A**). Western blot analysis revealed that the protein level of NLRP3 was higher in neutrophils from RA patients compared to those from OA patients (**Figures 6B, C**).

Moreover, we assessed NLRP3 expression in the synovial tissues of OA and RA patients. Compared to the OA group, RA patients exhibited increased levels of NLRP3 in the synovium (**Figure 6D**), which co-localized with MPO (**Figure 6E**). These results indicate an upregulation of NLRP3 expression in both peripheral neutrophils and synovial tissues of RA patients. Therefore, we hypothesize that the NLRP3 inflammasome may be involved in the pathogenesis of RA. This finding was further supported by immunofluorescence staining, which also demonstrated that NLRP3 expression in peripheral neutrophils was elevated in the RA group compared with the OA group (**Figures 8A, B**).

### 3.5 NLRP3 inflammasome participates in NETosis through GSDMD-N in RA

**Figure 7:**
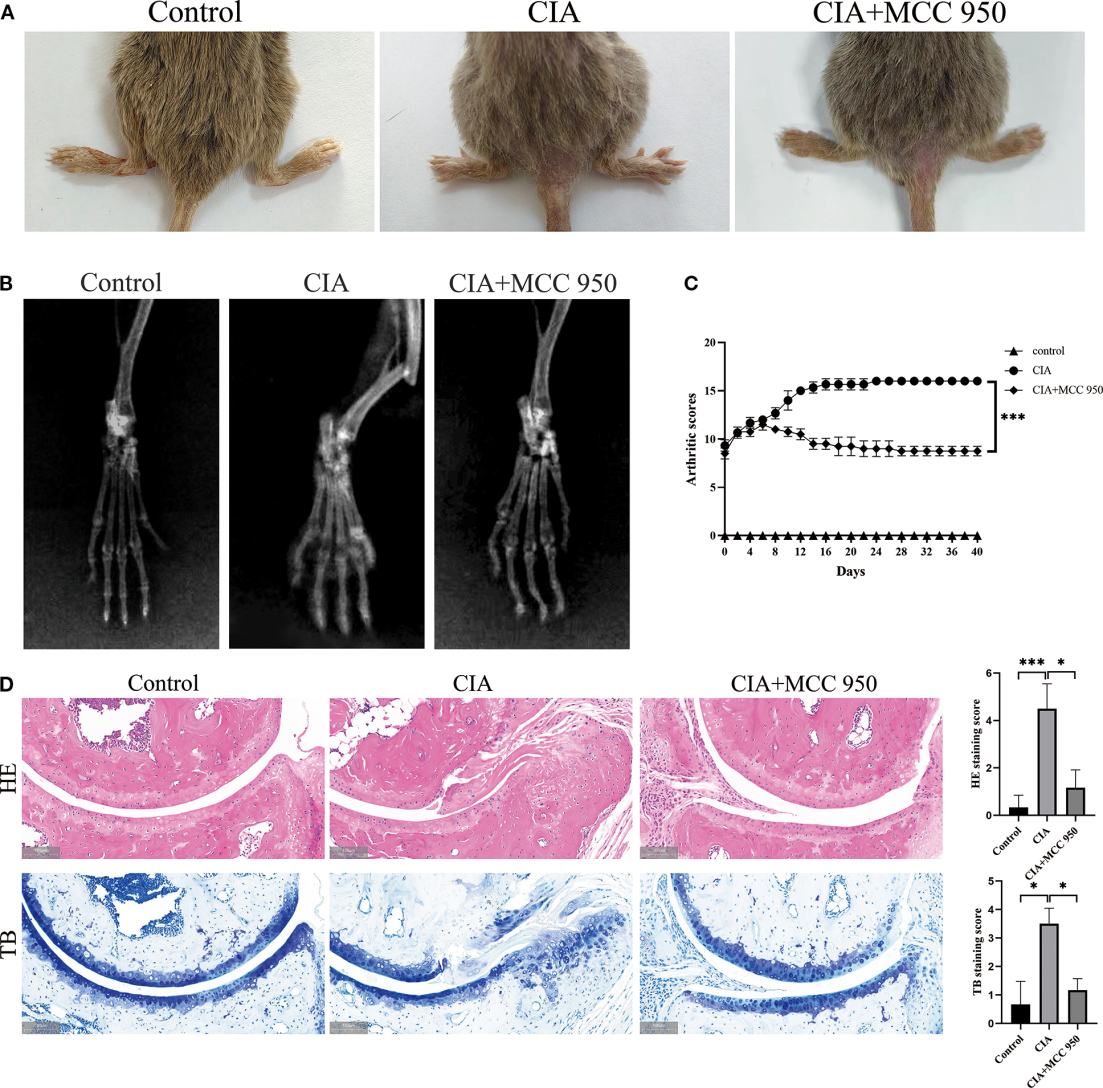
Inhibition of NLRP3 ameliorates collagen-induced arthritis (CIA) in mice. MCC950 was administered intraperitoneally to each mouse at 50 mg/kg every other day. **(A)** Representative images of hind paws from each group on day 40 of treatment. Arthritis severity was graded on a 0–4 scale. Data (mean ± SD, n = 6) compare the control, CIA, and MCC950-treated groups. **(B)** Micro-computed-tomography (micro-CT) cross-sections of hind-ankle joints after 40 days of treatment to evaluate bone destruction. **(C)** Arthritis scores for the mice. **(D)** HE and TB staining, along with histological scoring, were performed on ankle-joint sections collected on day 40 to evaluate synovial pathology and bone erosion/destruction.* p < 0.05, *** p <0.001.

Next, we established the RA model in CIA mice to observe whether NLRP3 has a significant effect on RA disease. After intervention with the NLRP3 inflammasome inhibitor (MCC 950), joint swelling and arthritis scores showed that inhibition of the NLRP3 inflammasome significantly improved the arthritis symptoms in the CIA group (**Figures 7A, C**). Radiographic evaluation of the ankle joints in CIA mice showed significant joint space narrowing and reduced bone density, while MCC 950 treatment significantly improved the radiographic features, alleviating joint space narrowing and bone density loss (**Figure 7B**). HE and TB staining images and score also showed that, compared to the CIA group, the MCC 950 treatment significantly reduced synovial cell infiltration and connective tissue proliferation covering the cartilage surface, and decreased soft bone and subchondral bone erosion (**Figure 7D**). These results indicate that inhibiting the NLRP3 inflammasome can alleviate the arthritis symptoms in CIA mice, which is consistent with our hypothesis that NLRP3 participates in the progression of RA.

**Figure 8:**
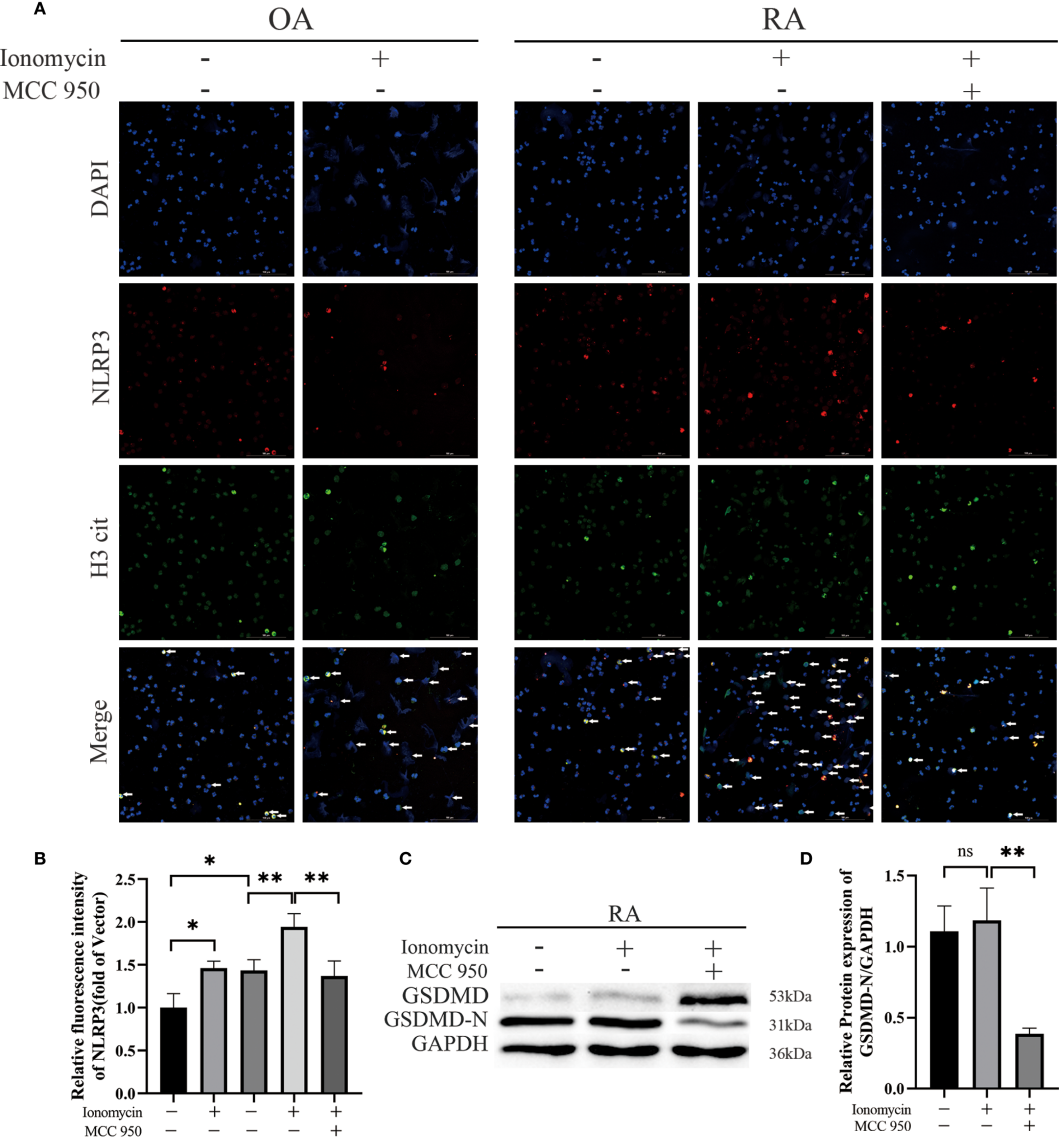
Effects of NLRP3 inflammasome inhibition on human peripheral-blood neutrophils. **(A)** Immunofluorescence staining was performed to assess the expression of NLRP3 and citrullinated histone H3 (H3 cit) in peripheral-blood neutrophils from patients. Neutrophils were stimulated for 2 h with MCC950 (10 µmol/L) or ionomycin (5 µmol/L). **(B)** Quantitative immunofluorescence analysis of NLRP3 expression in peripheral-blood neutrophils from OA and RA patients, with or without treatment. **(C, D)** Western-blot analysis and densitometry were used to compare the levels of activated GSDMD in peripheral-blood neutrophils after treatment with ionomycin (5 µmol/L) or MCC950 (10 µmol/L). * p < 0.05, ** p < 0.01, ns, not significant.

To further explore the specific mechanism by which NLRP3 contributes to the progression of RA, we stimulated peripheral blood neutrophils from OA and RA patients with ionomycin to induce NETs formation, and applied the NLRP3 inflammasome inhibitor MCC950 for intervention. Immunofluorescence analysis revealed that the levels of H3 cit were elevated in both the OA + ionomycin and RA + ionomycin groups. In addition, DAPI staining showed chromatin decondensation, indicating increased NETs formation upon ionomycin stimulation. Notably, inhibition of the NLRP3 inflammasome reduced ionomycin-induced NETs formation (**Figure 8A**). Subsequently, we performed Western blot analysis to examine the effect of NLRP3 inflammasome inhibition on GSDMD cleavage in neutrophils from RA patients. The results showed that inhibition of NLRP3 activation markedly decreased GSDMD activation, as evidenced by significantly reduced levels of the active N-terminal fragment GSDMD-N (**Figures 8C, D**). These findings suggest that suppression of NLRP3 inflammasome activation can reduce GSDMD activation and NETs release. Based on these results, we hypothesize that the role of the NLRP3 inflammasome in RA neutrophils may involve mediating GSDMD-N pore formation to regulate NETosis.

## 4 Discussion

In the early stages of RA, neutrophils accumulate in large numbers in the synovial fluid, releasing reactive oxygen species (ROS) and proteases, which directly damage joint tissues and exacerbate inflammation (1). Several studies have reported abnormal accumulation and increased activity of neutrophils in the synovial membrane of RA patients (1, 14, 15), and our experimental results are consistent with these reports. Furthermore, research indicates that GSDMD-dependent NETosis plays an important role in the progression of RA, and this process is activated by inflammasomes such as NLRP3, further promoting destructive inflammation in the joints (20). Therefore, abnormal neutrophil function and associated NETosis are considered to be significant mechanisms driving persistent inflammation and disease progression in RA.

NETosis can be induced by different factors and can be broadly classified into oxidative stress-dependent and non-oxidative stress-dependent NETosis. Oxidative stress-dependent NETosis is typically induced by pathogens such as PMA, bacteria, and fungi, and it requires ROS production. Oxidative stress-dependent NETosis is generally activated through the NADPH oxidase pathway. ROS plays a crucial role in this process, promoting the transport of MPO and NE into the nucleus to degrade chromatin. Non-oxidative stress-dependent NETosis, on the other hand, is induced by ionomycin or calcium ion channel agonists. This form of NETosis does not rely on ROS production but instead depends on an increase in intracellular calcium ion concentration to activate calcium-dependent signaling pathways, typically occurring more rapidly. Research by Gabriel Sollberger et al. demonstrated that membrane pore formation mediated by GSDMD-N is a necessary step for NET release (10). It regulates intracellular calcium ion channels, thereby inducing the release of neutrophil granules and chromatin decondensation, ultimately leading to NET formation. In this study, inhibition of GSDMD by Disulfiram in CIA mice significantly alleviated joint inflammation. Additionally, inhibition of GSDMD-mediated membrane pore formation in neutrophils from RA patients significantly reduced NET release. These findings indicate that GSDMD-mediated membrane pore formation is crucial for the release of NETs, and inhibiting this process can alleviate RA symptoms.

Through transcriptomic sequencing, we identified 12 differentially expressed genes (DEGs), including HSPA1A, HSPA1L, DNAJB1, HSPH1, HSPA6, HSPA14, HSPE1, HSPD1, H2BC17, H2BC7, H4C5, and H2BC8. These genes were significantly enriched in biological processes related to protein folding, chaperone-assisted protein refolding, and protein-DNA complexes, suggesting that protein folding and modification processes play essential roles in our study. KEGG pathway enrichment analysis further revealed that these genes are involved in immune and inflammatory-related pathways, such as cytokine-cytokine receptor interactions, chemokine signaling, and TNF signaling pathways. These results suggest that, in the process of NETosis, GSDMD cleaved into GSDMD-N can form pores in the cell membrane, leading to the release of cellular contents and the formation of NETs. This process is closely related to protein folding and cellular response pathways, particularly when neutrophils are damaged or activated. Cellular protein modifications and folding responses may regulate the activity of GSDMD and the formation of NETs. For example, heat shock proteins (HSPs) and DNA repair proteins displayed high enrichment in our analysis, and these molecules may indirectly regulate GSDMD activity by influencing protein folding, stability, and repair pathways, thus participating in the regulation of NET formation.

Among the 12 DEGs, HSPA1A, HSPA1L, DNAJB1, HSPH1, HSPA6, HSPA14, HSPE1, and HSPD1 are members of the heat shock protein (HSP) family, with HSPA1A, HSPA1L, HSPA6, and HSPA14 being part of the HSP70 family. Current research suggests that HSP70 is an essential molecular chaperone that promotes the correct folding of unstable proteins by binding to them, or directing them to the proteasome for degradation, to maintain intracellular protein homeostasis (21). Additionally, HSP70 can reduce oxidative damage by enhancing the expression of antioxidant enzymes, such as superoxide dismutase (SOD), and lowering intracellular ROS levels (22). DNAJB1, HSPH1, HSPE1, and HSPD1 are key proteins in the cellular stress response, maintaining protein homeostasis, assisting in folding, and removing damaged proteins to ensure normal cellular function (23–26). H2BC17, H2BC7, H4C5, and H2BC8 are histone variants, and existing research suggests they play specific roles in chromatin remodeling and gene expression, potentially participating in nucleosome formation and chromatin structure regulation (27). PPI analysis of these genes indicated potential interactions between HSPA1A and H4C5, as well as HSPD1 and H2BC17. In summary, we speculate that the inhibition of GSDMD pore formation, which reduces NET release, may be related to HSPs maintaining intracellular protein stability, reducing ROS levels, and influencing histone functions involved in chromatin structure regulation. Our research group is currently conducting further experiments to explore through which pathway Disulfiram suppresses NETosis by inhibiting GSDMD-N–mediated pore formation and inducing HSP70 upregulation.

Existing research indicates that GSDMD-N plays an important role not only in pyroptosis but also in NETosis (28). Pyroptosis is triggered by inflammasome activation (such as NLRP3), which cleaves GSDMD via caspase-1, leading to the release of pro-inflammatory factors (IL-1β, IL-18) and cellular contents. These substances amplify the inflammatory response and further aggravate tissue damage. NLRP3 is the most studied inflammasome, and current research suggests that in RA progression, NLRP3 is activated in response to danger signals both inside and outside the cell, leading to the activation of synovial fibroblasts, macrophages, T cells, monocytes, and other cells, resulting in pyroptosis (16) and ultimately contributing to synovitis and joint damage. In our experiment, we explored whether the NLRP3 inflammasome affects neutrophils in RA disease progression. Our results showed increased NLRP3 expression and co-localization with MPO in the synovium of RA patients. However, the role of NLRP3 in neutrophils is not yet fully understood. Our study showed that the expression level of NLRP3 in neutrophils from RA patients was higher than in those from OA patients. Some studies have reported that the NLRP3 inflammasome is highly activated in the synovium of RA patients and CIA mice, and this activation occurs mainly in infiltrating monocytes/macrophages within the synovium. The same study also pointed out that MCC950, can effectively alleviate arthritis symptoms in CIA mice (29). Our results also confirmed the therapeutic effect of MCC950 on CIA and further provided new evidence, demonstrating that MCC950 not only improves the clinical and histopathological manifestations of CIA, but may also exert its therapeutic effect by regulating NETosis in a GSDMD-dependent manner. This links NLRP3 inhibition to neutrophil-driven joint pathology and highlights its therapeutic potential in RA.

Previous studies have reported divergent mechanisms. Karmakar et al. reported that GSDMD-N in neutrophils does not translocate to the plasma membrane to induce pyroptosis but instead relocates to azurophilic granules, where it facilitates the release of MPO and NE, thereby promoting NET release. They further suggested that in neutrophils, the cleavage of GSDMD by caspases and/or serine proteases may depend on the strength or nature of the stimulus (9). Kaiwen W. Chen et al. proposed that the expression level of caspase-1 in neutrophils is relatively low and that the efficiency of caspase-1 in cleaving GSDMD is insufficient; therefore, caspase-1 in the canonical NLRP3 inflammasome is unable to effectively cleave GSDMD to generate GSDMD-N fragments that form pores in neutrophil membranes. They also noted that neutrophils do not resist cleavage mediated by the non-canonical inflammasome, and caspase-11 can trigger robust GSDMD cleavage and neutrophil NETosis (18). Patrick Münzer et al. demonstrated that in sterile conditions, the canonical NLRP3 inflammasome participates in NETosis, and during this process PAD4 can post-transcriptionally increase the levels of NLRP3 and ASC proteins, thereby facilitating NLRP3 inflammasome/ASC speck assembly and influencing inflammasome activation (19). Taken together, both canonical and non-canonical pathways of NLRP3 inflammasome activation are considered to be potentially involved in neutrophil NETosis, depending on the cellular context and type of stimulation.In our study, we observed GSDMD cleavage in peripheral blood neutrophils from RA patients, and intervention targeting GSDMD-N pore formation inhibited NETs release. Moreover, NLRP3 expression in the synovium and peripheral blood neutrophils of RA patients was higher compared with that of OA patients. Pharmacological inhibition of NLRP3 inflammasome assembly and activation with MCC950 markedly suppressed GSDMD cleavage, and a reduction in NETs release was also observed. In summary, we speculate that in the pathogenesis of RA, the NLRP3 inflammasome may participate in neutrophil NETosis by influencing the formation of GSDMD pores. However, this study did not examine ASC speck formation, caspase-1 activity, or the release of mature IL-1β/IL-18, which represents a limitation in drawing definitive conclusions. In our future experiments, we will focus on assessing ASC speck formation, caspase-1 activity, and mature IL-1β/IL-18 release to ensure the accuracy and robustness of our findings.

In our study, we used ionomycin to elevate intracellular Ca²^+^, a stimulus that is widely recognized to activate PAD4, promote histone citrullination, and drive chromatin decondensation, thereby inducing ROS-independent NETosis. The canonical NLRP3 inflammasome pathway proceeds through two steps: (I) priming, in which pro-inflammatory signals (e.g., TLR ligands such as LPS, TNF-α, and IL-1β) upregulate components of the inflammasome; and (II) activation, which can be triggered by ionic fluxes (K^+^ efflux, Ca²^+^ influx, Cl^-^ efflux), ROS, lysosomal rupture, and mtDNA release. These activation conditions partially overlap with those that promote NETosis.

In this study, we explored the role of the NLRP3 inflammasome in RA, particularly how it exacerbates the inflammatory response in RA through GSDMD-mediated NETosis. We detected GSDMD cleavage in peripheral blood neutrophils from patients with RA. When we induced NETosis with ionomycin, immunofluorescence demonstrated increased NET formation. Pharmacologic inhibition of NLRP3 with MCC950 [a selective NLRP3 inhibitor (30)] attenuated NET formation and was accompanied by a reduction in GSDMD cleavage. In summary, these observations support the role of the NLRP3–GSDMD axis in promoting disease progression in RA neutrophils through the enhancement of NETosis.

Transcriptomic analysis revealed that multiple genes related to protein folding and cellular responses were differentially expressed in the RA.Ion.D group, suggesting that these genes may indirectly regulate the activity of GSDMD-N by affecting intracellular protein stability, thereby modulating NET formation. In addition, we found that key genes, such as heat shock proteins (HSPs) and histone variants, may play an important regulatory role in suppressing GSDMD-N activity by influencing cellular stress responses and immune function. In summary, our study not only highlights the importance of the NLRP3/GSDMD pathway in RA but also provides new insights for future therapeutic strategies targeting this pathway. Future research could further explore the potential of inhibiting this pathway, offering new treatment options for RA patients.

## Data Availability

The raw data supporting the conclusions of this article will be made available by the authors, without undue reservation.
