# Supplementary material for: Analysis of GSDMD-N abnormality promoting neutrophil NETs mediated RA disease through NLRP3-dependent pathway
Source: Front Immunol. 2025 Sep 29;16:1652608. doi: 10.3389/fimmu.2025.1652608 (PMC12515664; doi:10.3389/fimmu.2025.1652608)
Supplement: Supplementary file 1 [file DataSheet1.docx]

Supplementary Table1 The baseline characteristics of OA and RA Patients

| Characteristics |  | | OA（n=30） | RA（n=30） | *P* value |
| --- | --- | --- | --- | --- | --- |
| Age (years, mean ± SD) | |  | 56.57 ± 6.95 | 58.43 ± 9.49 | 0.3883 |
| Sex (male/female) | male | | 5 | 7 | 0.747 |
|  | female | | 25 | 23 |  |

Supplementary Table2 Inclusion and Exclusion Criteria for OA and RA Patients

| **Criteria** | **OA** | **RA** |
| --- | --- | --- |
| Inclusion criteria | 1. Fulfillment of the ACR classification criteria for osteoarthritis. | 1. Fulfillment of the 1987 ACR or 2010 ACR/EULAR classification criteria for RA. |
|  | 2. ACR functional status class I–III. | 2. ACR functional status class I–III. |
|  | 3. No prior use of corticosteroids, DMARDs, biologics, or other immunosuppressive therapies before enrollment. | 3. No prior use of corticosteroids, DMARDs, biologics, or other immunosuppressive therapies before enrollment. |
|  | 4. Patients had a full understanding of the experimental principles and objectives and voluntarily agreed to participate in this study. | 4. Patients had a full understanding of the experimental principles and objectives and voluntarily agreed to participate in this study. |
| Exclusion criteria | 1. Coexisting autoimmune or rheumatic diseases (e.g., rheumatoid arthritis, ankylosing spondylitis, systemic lupus erythematosus). | 1. Prior treatment with corticosteroids, DMARDs, biologics, or other immunosuppressive therapies. |
|  | 2. Acute or chronic infections, malignancies, severe cardiovascular, hepatic, renal, or hematological disorders. | 2. Coexisting autoimmune or rheumatic diseases (e.g., systemic lupus erythematosus, Sjögren’s syndrome, ankylosing spondylitis, or gout). |
|  | 3. History of joint replacement surgery or intra-articular corticosteroid injection within the past 6 months. | 3. Acute or chronic infections, malignancies, severe cardiovascular, hepatic, renal, or hematological disorders. |
|  | 4. Pregnant or lactating women. | 4. Pregnant or lactating women. |
|  | 5. Inability or unwillingness to provide informed consent or comply with study procedures. | 5. Inability or unwillingness to provide informed consent or comply with study procedures. |

Supplementary Table3 Top 20 in network Sheet 1 ranked by MCC method

| Rank | Name | Score |
| --- | --- | --- |
| 1 | HSPA8 | 43592542 |
| 2 | HSPA1B | 43592462 |
| 3 | HSPA2 | 43591770 |
| 4 | HSPA1A | 43591704 |
| 5 | HSPA1L | 43591680 |
| 6 | HSP90AA1 | 43587691 |
| 7 | DNAJB1 | 43586784 |
| 8 | HSPH1 | 43586064 |
| 9 | HSPA6 | 43551414 |
| 10 | HSPA14 | 43550640 |
| 11 | HSPE1 | 39917664 |
| 12 | HSPD1 | 39916951 |
| 13 | H3C13 | 3991798 |
| 14 | H2AZ1 | 3991783 |
| 15 | H3C6 | 3991740 |
| 16 | H2BC17 | 3991704 |
| 16 | H2BC7 | 3991704 |
| 16 | H4C5 | 3991704 |
| 16 | H2BC8 | 3991704 |
| 16 | H2BC15 | 3991704 |

Supplementary Table4 Top 20 in network Sheet 1 ranked by MNC method

| Rank | Name | Score |
| --- | --- | --- |
| 1 | HSP90AA1 | 29 |
| 2 | HSPA8 | 24 |
| 3 | H3C13 | 22 |
| 4 | HSPA1B | 21 |
| 5 | HSPA2 | 20 |
| 6 | H2AZ1 | 17 |
| 7 | HSPA1A | 16 |
| 7 | HSPA6 | 16 |
| 9 | DNAJB1 | 15 |
| 9 | HSPA1L | 15 |
| 9 | H3C6 | 15 |
| 12 | HSPE1 | 14 |
| 12 | HSPD1 | 14 |
| 12 | HSPH1 | 14 |
| 15 | H2AX | 13 |
| 15 | HSPA14 | 13 |
| 17 | H2BC17 | 12 |
| 17 | H2BC7 | 12 |
| 17 | H4C5 | 12 |
| 17 | H2BC8 | 12 |

Supplementary Table5 Top 20 in network Sheet 1 ranked by DMNC method

| Rank | Name | Score |
| --- | --- | --- |
| 1 | H2AC13 | 0.90 |
| 1 | H2AC17 | 0.90 |
| 3 | HSPA14 | 0.89 |
| 4 | HSPH1 | 0.86 |
| 5 | HSPA1L | 0.82 |
| 6 | H2BC17 | 0.82 |
| 6 | H2BC7 | 0.82 |
| 6 | H4C5 | 0.82 |
| 6 | H2BC8 | 0.82 |
| 6 | H2BC15 | 0.82 |
| 6 | H4C3 | 0.82 |
| 12 | DNAJB1 | 0.81 |
| 13 | BAG3 | 0.80 |
| 14 | POM121 | 0.77 |
| 15 | HSPA1A | 0.76 |
| 16 | HSPE1 | 0.75 |
| 17 | HSPA6 | 0.73 |
| 18 | HSPD1 | 0.72 |
| 19 | SERPINH1 | 0.71 |
| 20 | H2AX | 0.64 |
